# Lanfranc of Milan

**Published:** 1873-12

**Authors:** James Nevins Hyde

**Affiliations:** Chicago


A MONTHLY RECORD OF

*Medicine, Surgery and the Collateral Sciences.*

EDITED BY J. ADAMS ALLEN, M.D., LL.D.; AND WALTER HAY, M.D.

---

VOL. XXX. — DECEMBER, 1873. — NO. 12.

---

### Original Communications.

ARTICLE I.—*Lanfranc of Milan. His Brief. Reduced to our Vulgar Phrase.* By JAMES NEVINS HYDE, M.D., Chicago.

The little volume before me is entitled: " A most Excellent and Learned Woorke of Chirurgerie, called, *Chirurgia Parva Lanfranci.* Lanfranke of Mylayne, his briefe, reduced from dyvers translations to our vulgar or usuall frase, and now first published in the Englyshe prynte by John Halle, Chirurgien. Who hath thereunto necessarily annexed A Table, as wel of the names of diseases and simples with their vertues, as also of all other termes of the arte opened. Very profitable for the better understanding of the same, or other like workes. And in the ende a compendious worke of Anatomie, more utile and profitable then any here tofore in the Englyshe tongue published. An Historiall Expostulation also, against the beastly abusers both of Chyrurgerie and Phisicke in our tyme. With a goodly doctrine and instruction, necessary to be marked and folowed of all true Chirurgiens. All these faithfully gathered and diligently set forth by the sayde John

Halle. Imprinted at London in Flete streate nyghe unto saint Dunstone's churche by Thomas Marshe, An. 1565."*

It is difficult to scan the distinct Black-Letter, impressed three hundred years ago upon these yellow pages, without feeling an interest in the man whose thoughts are thus conveyed to us across the arch of time. Who was he? Under what circumstances did he practice that profession, whose stores of learning have been garnered and transmitted for the use and profit of to-day?

> ". . . . Si quæris vultum dignoscere verum
> Hos lege. Hii vere explicuere animum ;"

Reads the text beneath the engraving that represents to us his features.† This latter is crude enough. But he will not wonder overmuch, who reflects that it was completed only ninety-four years after the first book printed in England, issued from the first English press; and that forty years elapsed subsequently before the formation of a school of English engravers. The frontispiece shows us an honest, square, full-bearded, Saxon face, beneath a skull cap and hat not unlike that now worn by the warders of the Tower of London. The broad high collar and facings of the coat are apparently of taffeta. In the left hand is held one of the simples referred to in the title-page.

There is nothing in the portrait which would preclude the idea that the author of the work possessed also such physical gifts as are described in his preface as "the properties of a chirurgien: He shoulde bee of cleare and perfecte sighte, well formed in person, hole of minde and of members, sclender and tender-fingred, having a softe and stedfast hande, or, as the common sentence is, a chirurgien shoulde have three dyvers properties in hys person, that is to saie, a harte as the harte of a lyon, his eies like the eies of an hawke, and his handes as the handes of a woman." Were these the "properties" of all the surgeons of the 19th century, their fame and features might be preserved for as many hundred years as those of Maister John Hall.

* This rare and curious specimen of the medical literature of the 16th century is the property of Dr. Charles Gilman Smith, of this city, and was recently procured by him of an antiquary in London.

† This engraving has been several times copied as an interesting evidence of the infancy of the art at that period. It is very accurately reproduced in Sargent's Collection.

The England in which our author "diligently set forth his compendious worke," was far different from the England of this decade. That England awaited the dawning of a light that was to enliven science, literature, art and religion with its vivid beams. But the year 1565 knew only of the darkness that precedes the dawn, although a few struggling rays had already brightened its horizon. The glory of the reign of Elizabeth had yet to be declared. The eyes of the baby, William Shakspeare, looked out wonderingly upon the placid waters of the Avon. Protestantism and Catholocism were alike intolerant and aggressive. Ten years before, Hugh Latimer had walked to the stake with Coverdale's bible clasped to his girdle. The author of the Faerie Queene was a student twelve years of age. In Italy, Galileo had but just signed his renunciation of the Copernican theory. French was the language of the English court and nobility. English was indeed, as our author terms it, "the vulgar or usuall frase," and was read or written by few of those who spoke it. Surgeons were still licensed to practice by the Bishops of the Established Church—a fact indirectly confirmed by the text before us:

" . . if none but the learned and experte myght have wrought and they also admitted by the byshop: as it hath in tymes past been holsomlye ordeyned in this Realme."

This responsibility was, however, imposed soon after this date upon the College of Physicians of London, which was founded by Thomas Linacre, of Canterbury, physician to Henry the VIII, the Queen's father. Linacre was himself also an author, and published the first work in the Latin language which was issued from an English press. He also established a chair of Hippocratic and Galenical medicine in the Universities of Oxford and Cambridge.

It is evident that some organization of the regular practitioners of surgery, in London and its vicinity, had been at this time effected.* The "Epistle Dedicatorie," of the volume before us, is "Unto the Worshipful, the maisters, wardens, and consequently to all the whole company and brotherhod of Chirurgiens of London," wherein they are bidden to "bee thankefull to maister William

* The Company of Barbers of London, and the Company of Surgeons of London, were united in the year 1540, under the title of the "Masters and Governors of the Mystery and Commonalty of the Barbers and Surgeons of London." The Company of Surgeons of London dates from the year 1511.

Cunningham,* doctor of phisicke, for his so many learned lectures which be red in our halle." Doctor Recorde had, it appears, dedicated his "Urinale of Phisicke" to the same body; and Doctor Turner† had "compyled his englyshe herbale for their sakes."

The maister Cunningham alluded to, is the author of a letter which succeeds the dedicatory epistle, commending highly "unto the professors of Chirurgerie in London, the labours of John Halle, one of theyr fellowshippe," and in which he expresses a doubt "whether Lanfranke oweth more to him [Hall] for the restoring of his decayed worke, or he to Lanfranke for the immortall fame hereby obteined." This is followed by a letter from Thomas Gale,‡ "maister in Chirurgerie, to his well beloved friende Halle," written evidently with a view to further the adventurous scheme of publication. Its encomium upon the art of surgery is forcibly expressed: "for, without it, pleasures were but painful, riches unprofitable, company annoyance, strengthe febelnes, bewtie lothsumnes, senses dispersed, and finally all things in the worlde unpleasant."

We have, then, internal evidence of the fact that Hall was not only highly esteemed by eminent contemporary authors, who were his friends, but was himself also a surgeon of repute. He was born in 1529, and, at the age of thirty-five years, was engaged in the practice of his profession in Maidstone, Kent. Bishop Tanner states that he wrote and translated several other surgical treatises. We learn also, from the same source, that he subsequently composed a volume of hymns. Cunningham tells us that "he hath also framed an other worke, inveying against vice, and therefore named the *Court of Vertue*, being now in the printer's handes."

* Cunningham (or Cunyngham) was, at the age of twenty-eight years, a physician of Norwich; and there is an extant engraving, representing him with Dioscorides' book of plants in his hand. He was the author of several works. One is entitled the "Cosmographical Glasse, conteyning the pleasant principles of Cosmographie, Geographie and Hydrographie or Navigation." Another treatise has for its subject the French Disease, or Syphilis of our nomenclature. Still another is a commentary on the works of Hippocrates.

† Dr. Turner's Herbal, here noticed, was published in 1551, as a Black-Letter folio, and is the oldest Herbal in the English language.

‡ Thomas Gale, or, as he sometimes wrote his name, Galus, was one of the surgeons of Queen Elizabeth. One of his works is dated in the year 1563.

His efforts in verse, which are scattered along these pages, though exceedingly puerile when criticized in the purview of modern poetry, yet compare favorably with the stanzas of the Battle of Chevy Chase, which was the popular ballad of his time. His medical library could hardly have contained many volumes beside the published productions of his friends, already enumerated, with perhaps "Bulleyn's Bulwarke of Defence against all sicknes, sorenes and woundes that doe daily assault mankinde," published in 1562; and possibly also a few Latin and Greek commentaries on the medical teachings of the classic and Arabian schools.

In the introduction to the treatise of Lanfranc, he states that the whole art of medicine is contained in three principal parts: Physiology, (which is made to include Anatomy), Pathology, and Therapeutics. The latter, or curative, part is described as having three instruments: Dietetics, Pharmacy, and Surgery. After remonstrating against the tendency, then prevailing, to a complete separation of these branches of medicine, he concludes by detailing the reasons which led him to reproduce in English the work of the Italian master.

Lanfranc was born in Milan, but was compelled to leave his native city in consequence of some political difficulties. He went to Paris in the year 1295, where he was known as a "medecin chirurgique"—a term then applied to doctors in medicine who practiced surgery.* He soon became the first of a long line of brilliant surgeons who have illustrated the genius and skill of the French in scientific pursuits. Pupil of Guillaume de Salicet of Placentia, he had readily surpassed his preceptor in the boldness and success of his undertakings. His work entitled Grande Chirurgerie, was the most authorative compilation of surgical science up to the conclusion of the 14th century, and we are not surprised to learn that the English surgeons of the 16th century studied and translated his writings with the reverence of scholars for the treasures of antiquity. With them, antiquity was equivalent to authority.

The treatise selected for publication by Hall, is the "Chirurgia Parva," or minor work. We are informed that it was originally composed in the form of a letter to a friend, as a prelude to a greater work subsequently published in 1296. This greater work is, without doubt, the "Grande Chirurgerie." The letter,

* Dunglison's Hist. of Med. Phil., '72.

or brief, is addressed by Lanfranc to his "faithfull frende and disciple Bernard," and was probably written, as the Italian and French titles seem to indicate, before his resort to Paris.

The limits of an article in the JOURNAL, preclude the possibility of making extended extracts from these quaint dissertations. Forty-eight pages only are allotted to the text proper, and twenty-four to the annexed "Antidotarie." The subject of fractures is exhausted in twenty-seven lines; that of dislocations occupies merely one page and a half. A portion of the seventh chapter, entitled, "Of the Wounde of the Heade with breakinge of Cranion," is interesting as an indirect confirmation of the idea that there is nothing new under the sun, and that the practice of auscultation and percussion was recommended five hundred years before the birth of Laennec:

"When the wounde is made in the heade with breakinge of the sculle, consider whether it be broken unto the inwarde partes or no, that is to saye, to *duram matrem*, whiche thou maiste knowe by divers meanes and waies, partelye by perseverance and partelye by infallible experimentes.* The signes be these: the feelinge of greate paine, vomiting, teares of the eies, crokednesse of the syghte, inflammation or rowlinge of the eies. And the experimentes are these: take a stronge threde, double twisted and wexe it, and let the patiente holde it stronglye in his tethe, and begin thou at the mouthe of him, and with thy nailes stretche and streigne oute the threde, til thou come at the other ende of the same, holding it streight a cubite lengthe from the tethe, and make a sounde upon the threde with thy nayle, and doe so often times. If the patiente may susteine the sounde without feelynge of peine, then is not the sculle broken to the *dura mater*, for, if it be broken, he maye in no wise susteine nor suffer the harping of the nayles upon the threde. Or else thou mayst also take and smyte his head with a smalle dry wand of wylowe, or of y[e] pine tre, and *holde thine eare* to hys head. And if the sculle be whole, it wyle make an hole sound: but if it be cutte or broken, it will make a dumme noyse, after the comparison of a broken bell and a whole."

* These pages supply abundant evidence of the fact that the English language was at this time in its formative stage. The old black letters, or Gothic type, were originally *fac similes* of the characters found in manuscript. The various spelling of the same words in one paragraph, probably occurred in, and was copied from, Hall's chirography.

The treatment advised is, the enlargement of the wound, if the fractured surface be more extensive than the external lesion, and the removal of any loose pieces of bone, which may be encountered. If depression of the bone has occurred, the measures to be pursued are by no means heroic. Warm oil of roses—an application to wounds evidently much esteemed by the author—is to be infused, and small pledgets of tow or lint superimposed, soaked in the same preparation with the yolk of an egg added, hot. On the second or third day thereafter, an effort is to be made "by most tender and delycate workinge wyth convenient instrumentes," [not otherwise described],* to remove, not elevate, the depressed portion. The membranes were to be carefully avoided, and the operator is to have in mind "the lytell shiver that greueth the *dura mater*."

The external wound was to be kept open till the fracture was healed, by the aid of lint or tow, saturated with the lotion described, and over the whole were to be "bounde greate boulsters or pressures of towe, wete in the somer time in colde water, and in the winter in whotte water."

The second part of the work treats of "Apostemes." Hall declares that "this word signifyeth the same whyche the Latines caule *abscessum*." There are chapters on watery apostems, and windy apostems, apostems of natural and unnatural humors, hot, cold and compound apostems. It is a convenient word. Under it are discussed disorders of the most diverse etiology, including icterus, cutaneous diseases, carbuncles, and quotidian fevers. In the appended "Antidotarie" we find many familiar names of medicaments, interspersed with others that are strange and disgusting. Of the former class are, manna, scammony, aloes, lactucarium, opium, hyosciamus, cardamom seeds, cubebs, anise, chamomile, yeast, flaxseed, and the axangia porci.

It must be confessed that the anatomical compend by Hall, which succeeds the translated portion of his work, scarcely betrays the shadow on the dial plate of the three hundred years that divide them. It furnishes abundant proof that dissections of the human body were as yet neither permitted nor practiced in England. Mondini de Luzzi, in the year 1315,† dissected the human

* The trephine was first employed by Fabricius ab Acqua pendente at the close of the 16th century.

† Renouard, Hist. of Med.

cadaver in Bologna for the first time for seventeen centuries, and the acquisitions obtained through his anatomical studies, sufficed the world for nearly two centuries longer. It is exceedingly doubtful whether Hall and his associates, who quote for their authorities Galen and Hippocrates, were informed regarding Mondini's investigations. The international communication of their period, was had in the interest of sovereigns and their servants, rather than of savants. From his silence on that subject, we may, for example, conclude that Hall knew nothing of the labors of Ambrose Pare in Paris, who yet at this very time, published an account of the pathology and treatment of gunshot wounds produced by arquebuses—a species of injury then novel to the surgeon and the soldier.

Four anatomical plates only are appended, and these represent exclusively the external surfaces of the body. The explanations of the cuts are connected by lines with the parts named, but nowhere is the site of viscera or muscles indicated. There is one feeble attempt to outline the bones. The student is referred merely to the nares, the venter, the genæ, the pectus, etc. The plates themselves are so rude that they bear a curiously suggestive resemblance to those which illustrate a Chinese medical work in the library of the writer of these pages. For comparative excellence the palm should certainly be awarded to the edition of the Celestials.*

It must not be forgotten that the circulation of the blood was not discovered by Harvey till fifty-seven years after the date of Hall's publication; and that the latter still believed in the existence of the four animal humors: blood, phlegm, choler, and melancholy. His practice was based chiefly upon the four temperaments which were considered to result from these humors, viz.: the sanguine, the phlegmatic, the choleric, and the melancholic.

The chapter, "Of the hearte," is subjoined in full, as a sample of his other anatomical descriptions:

"And wythin those bones, that is to saye, the bones of the brest, the rybbes and the spondilles of the same, wythin the holownesse that is made of them, I saye, is the Hearte, named in Greeke,

* This volume was procured in Shanghae, by Dr. C. D. Maxwell, U. S. N. The plates are probably reproduced from some old compend of medicine in the Portuguese language.

*cardia*, and in Latyne, *cor*, confyrmed and sette. Whyche, because he is the principall member of all other members, and the beginning of life, is thus sette in the myddest of the breaste, as lorde and Kinge to all the rest: of whome he is obeyed and served, as a prince of hys subjectes. And the hearte hath bloude in hys owne substance, wheras all other members have it but in arteries and veines: and in the hearte is the nutrityve bloude made livelye spirite, and carried forth in the arteries, whiche in the hearte have theyr beginnynge. And the hearte is covered with a strong pannicle, called of the Latines, *capsula cordis*, and of the Grecians, *pericardion*. And from the hearte procedeth the greate arterie, whiche is called in Latine, *arteria magna*, from whom brauncheth and procedeth all the other arteries y[t] are in anye member of the bodye, by which meanes the spirite of life is caried to all the members of the same. And the hearte is an offyciall member, spermatike, and of a lacertous substance. The greate ende wherof in his being leaneth and inclineth moste unto the ryghte side, and the small ende leaneth moste unto the lefte side. And in the hearte have the venall arterye and the arteriall veine theyre begynninges, of whose processes and offices I speake immediatlye hereafter in the lunges. Here also mighte be to greate purpose declared the greate secretes of these firste movinges whiche are in the hearte, called in greke, *systole* and *diastole*, in latine, *contractio* and *dilatatio*, which are compared in this orbicle (of divers learned men) to the *primum mobile*, or firste mover in the greate orbe. For these movinges are the first cause of all other movinges, as of pulses in the arteries and so of all the rest. But because these secretes passe the capacitye of the comon sorte, and also that I shoulde breake my purpose of briefnes, I omit them."

[O maister Halle, moste admirable is thy ingenuitye!]

It is clear that the county seat of Kent, where Hall was engaged in practice, was as much the resort of empirics as the larger cities of our own country. The indignation and disgust of our author at the practices of these folk, is meted out on almost every page of his book, in no stinted measure. He evidently could not tolerate an intrusion upon the fair domain of his art, by the "beastlie abusers both of phisicke and chirurgerie." His "historiall expostulation" is an honest and manly protest against the laxity of the laws which permitted the impositions of men and even women, who enticed and deluded the common people with astrology, divination and magical remedies.

It is a curious fact that both he and Lanfranc hesitated to publish their works because they were fearful lest the ignorant should, by ever so little understanding of the contents, prostitute the results of their labor to unworthy ends. The Italian charges his friend Bernard to deliver his letter to "no rusticall Fooles or Idiotes, leaste by the ignorance of such, and by theyr rude and uncunninge ministration, this my worke (made for thy love to a common utilitye) myghte redounde to some personnes hurte or annoyaunce." And the Englishman is "not ignorant that some will thynke that this booke (beyng published) wyll be an occasion for suche men to be the bolder to abuse the same science. But I knowe and am sure that they shall not learne in this booke any thinge wherwith to hurte. Neither have I published the same for them. And further, if any abuser of Chirurgery reade this boke, he shall, I trust, so finde hymselfe rubbed on the galle, that he shall be moved (if he have any shame) to leave his vice rather than more to use it."

Unfortunately, such men have now no more shame, than when these words were first penned for their edification. The details furnished of their performances in Maidstone, sound strangely like those which at intervals find their way into our public journals. There was, for example—

". . . One Robert Nicols, a false deceiver and moste ignorante beaste and of the profession of vagaboundes. Who boasted him selfe to have been the servaunte of the late Sergeant Chirurgien to the Quene's highnes. But now the matter being put in triall, he sayeth he was apprentice with a priest. Among whose wicked and prodigious doynges (which are infinite) one very notable chaunced in the yere of our lorde, 1564, the 26 of September. He poured in a purgation to an honest woman of good fame, one Riches, wydowe of Linton, a parysh three myles distant from Maydestone, whiche within three or foure houres at the moste, purged the lyfe out of hyr body, so violente was this mortal potion. The woman being before in perfecte health to all men's judgementes: being onely of simplicitie persuaded to take the same, by the deceivable perswasions of this Nicols. Who made fayre wether of all thynges, and hir to believe that he would deliver her of such diseases as indeede she had not." We are not surprised to learn that the unfortunate woman had been told by the imposter, that "hyr lunges were rotten."

The volume concludes with a species of Code of Ethics in metre,* that is fully as admirable as that adopted by our own National Association, since it is based upon the only golden rule of professional conduct. There are fifty-four verses in all. I select two as examples of the rest :

> "See that thou laye disdeyne aside,
> And pride of thyne own skyll :
> And think no shame counsell to take
> But rather with good wyll."

> "Sithe Bernarde knewe not all hym selfe
> Thinke never in thy minde,
> But that at laste, by painfull proofe
> Thou shalte all erroures fynde."

The study of the medical lore of past centuries is calculated to inspire a feeling of contempt or admiration, according to the spirit in which it is conducted.

Some, for example, can thereby merely accumulate proofs of the immense superiority of modern researches. The Cilician tent-maker wrote from Ephesus to Corinth, in A. D. 57,† that there was "one kind of flesh of men, another flesh of beasts, another of fishes, and another of birds." In 1868, a member of the Royal College of Surgeons of London,‡ found a "community of protoplasm in the finner whale, disporting his eighty or ninety feet of blubber with an easy roll amid waves in which the stoutest ship would founder, and the microscopic fungus, finding space enough to multiply into countless millions in the body of a living fly." Between these two men there is, for some, a deep which they cannot fathom.

* If this production appear ridiculous in the eyes of any reader, let him consult the chess-problems, theatrical criticisms and society gossip, to be found in Vol. I of the *London Lancet*, published in 1824.

† I Cor. 15 : 39.

‡ "On the Physical Basis of Life." T. H. Huxley, LL.D., F.R.S. *Fortnightly Review*, 1869.

Another,* who justly deserves to be named the Chrysostom of medical authors, speaks in this wise: "Our ancestors, less rich than we in the knowledge which it is our privilege to utilize, devoted themselves incessantly to the labor of production. Though poor in facts, they put to the proof every fragment which chance or experience brought within their reach, as the athletes who exercise their muscles, and there resulted a force, which, though it may have occasionally betrayed itself by remarkable perversion, was yet gifted with an insight full of grandeur and prolific in value. Their efforts multiplied in proportion to the poverty of their resources, and their success was astonishing. While we, whose opportunities are unbounded, are enervated, relaxed, spoiled by the very abundance which is offered to us. We are capable only of receiving and becoming engorged. Our intelligence is oppressed with satiety, and perishes unproductive."

Is there no nobler lesson to be read in the palimpsests of medicine? The Rig-Veda indicates that the language spoken before the birth of the Aryan and Sanscrit, recognized the relations of husband and wife, brother and sister, that subsist in the English homes of the present.† Viewed from that eminence which all science, by virtue of its immortality, aspires to reach, there is a wonderful kinship between Buddha, renouncing his palace and his princedom "in order to make captive old age, disease and death," and the observer, who in 1873, describes the cryptogamic spores that yield a harvest of malaria.‡

The aims, disappointments, labors and triumphs of John Hall, and his fellow-chirurgiens, are ours to-day. And who can read the apostrophe that closes his little volume without a conviction that he knew of the phases and problems of human life, which are the same in succeeding ages of the world:

"The bodye of man is called in Greeke, *microcosmos*, that is to saye, a lyttle world.§ For it is a lyttle world in deede, to be con-

* Trousseau. Introd. Clin. Med. de L'Hot. Dieu de Paris, 1865.

† Comparative Mythology. Muller, 1856.

‡ Dr. John Bartlett, of this city, now convalescent after an attack of remittent fever, has discovered in his blood and saliva the sporules of the palmellæ, planted purposely in his sleeping apartment.

§ Mayhap Sir Thomas Browne, Kt. M.D., had read this page when he wrote in 1640: "I take my circle to be above three hundred and sixty: though the sidered in order of Anatomye, yea, a wonderfull worlde, such a worlde in dede as the greate worlde was made for the onlye sake of it. And as some have named it a lyttle worlde, so have other some called it a common weale, a royall kingdome. For in this common weale was never found rebellion, but every subjecte servisable to his governor, and every superior tendering the wealth of his inferyors. Oh worthy common weale, by whom all common weales may take sufficient example and vewe, howe to governe or to be governed! Oh wilfull and negligent man y[t] runnest a stray as one not knowinge the rule of right! What nedest thou a preacher in such a case, where thou art sufficientlye taught, if thou wilte learne in thyne owne creation! Oh whye shoulde that creature be brute, or insypient, or as a salvage beast, whiche for his wonderfull giftes, geven him of God in hys creation, is called a lyttle worlde or a common weale! What a strange thinge were it that he whyche is a common weale in hym selfe, and carieth continually wythin hym selfe the perfect image of a common weale, shoulde, in a common weale, bee a cause of a common destruction."

